# A Multi-Class ECG Signal Classifier Using a Binarized Depthwise Separable CNN with the Merged Convolution–Pooling Method

**DOI:** 10.3390/s24227207

**Published:** 2024-11-11

**Authors:** Rui Zhang, Ranran Zhou, Zuting Zhong, Haifeng Qi, Yong Wang

**Affiliations:** School of Integrated Circuits, Shandong University, Jinan 250101, China; ruizhang2022@mail.sdu.edu.cn (R.Z.); zuting_zhong@mail.sdu.edu.cn (Z.Z.); hf_qi@sdu.edu.cn (H.Q.)

**Keywords:** binarized depthwise separable convolutional neural network (bDSCNN), ECG, blockwise incremental calculation, merged convolution–pooling method, multi-classifier, FPGA

## Abstract

Binarized convolutional neural networks (bCNNs) are favored for the design of low-storage, low-power cardiac arrhythmia classifiers owing to their high weight compression rate. However, multi-class classification of ECG signals based on bCNNs is challenging due to the accuracy loss introduced by the binarization operation. In this paper, an effective multi-classifier system is proposed for electrocardiogram (ECG) signals using a binarized depthwise separable convolutional neural network (bDSCNN) with the merged convolution–pooling (MCP) method. The binarized depthwise separable convolution layer is adopted to reduce the increased number of parameters in multi-classification systems. Instead of operating convolution and pooling sequentially as in a traditional convolutional neural network (CNN), the MCP method merges pooling together with convolution layers to reduce the number of computations. To further reduce hardware resources, this work employs blockwise incremental calculation to eliminate redundant storage with computations. In addition, the R peak interval data are integrated with P-QRS-T features to improve the classification accuracy. The proposed bDSCNN model is evaluated on an Intel DE1-SoC field-programmable gate array (FPGA), and the experimental results demonstrate that the proposed system achieves a five-class classification accuracy of 96.61% and a macro-F1 score of 89.08%, along with a dynamic power dissipation of 20 μW for five-category ECG signal classification. The hardware resource usage of BRAM and LUTs plus REGs is reduced by at least 2.94 and 1.74 times, respectively, compared with existing ECG classifiers using bCNN methods.

## 1. Introduction

According to the World Health Organization, cardiovascular diseases (CVDs) are the leading cause of death, having been estimated to cause 17.9 million annual deaths globally [1,2]. Thus, the detection of CVDs in their early stages can reduce later complications and save curative costs [3,4,5]. Unfortunately, early-stage CVDs usually have no obvious symptoms [6,7] and are easily overlooked. Recent research has shown that detecting early-stage CVDs using electrocardiogram (ECG) sensors [8,9,10,11] provides a feasible solution to realize real-time monitoring and can decrease death rates effectively. As a result, the development of wearable devices for ECG signal detection and classification has become a trend and is attracting more attention [12,13,14,15,16,17,18,19].

Limited by local data processing capability, early versions of wearable ECG monitoring devices transmit raw ECG data to health centers via wireless networks [14,20]. Although these central processing approaches can achieve high detection accuracy, continuous data transmission often consumes noticeable power, thus necessitating frequent battery recharges. In recent years, developing artificial intelligence (AI) techniques have provided an alternative way to detect heart arrhythmia on the spot [21,22,23,24,25,26,27,28,29,30]. For example, an adaptive 1D convolutional neural network (CNN) was deployed to realize feature extraction and classification for five-class ECG signals [23]. Moreover, a fuzzy neural network with wavelet transform [24] and a two-stage neural network [25] were proposed to detect premature ventricular beats. Also, a classifier with support vector machine (SVM), random forest (RF), and k-nearest neighbors (KNN) was presented to identify inter-patient atrial flutter [26].

Despite the above achievements, characteristics associated with traditional AI, such as multiple parameters and complex operations, still require noticeable hardware resources and power consumption, making its application a challenge in medical edge computing scenarios. Several recent works have focused on hardware implementation [31,32,33,34,35,36,37] to improve the efficiency of ECG classifiers. For example, a hybrid architecture consisting of long short-term memory (LSTM) cells and multilayer perceptrons (MLPs) was realized in an embedded device for ECG binary classification [31]. A lightweight spiking neural network (SNN) model was implemented on a field-programmable gate array (FPGA) platform to realize a five-classifier for ECG signals [35]. Another five-classifier was designed as an application-specific integrated circuit (ASIC) by using an artificial neural network (ANN) structure [36]. Although these systems can classify two or more types of ECG rhythms with relatively high accuracy, they often involve complex operations, such as numerous multiplication with floating-point or n-bit fixed-point operations, which could be simplified for better power performance.

Considering the hardware resources and the power constraints of edge biomedical devices, using fewer bits for neural networks is desirable when the accuracy requirement permits. As an extreme case, a binarized CNN (bCNN) is expected to have the most concise format [38,39,40,41,42,43,44]. Due to the reduced bit width, a bCNN requires significantly lower memory bandwidth and less memory storage compared with its multi-bit CNN counterpart [45]. Several works have shown that bCNNs can achieve reasonable classification accuracy and high energy efficiency for binary classification of ECG signals [46,47,48]. For instance, a bCNN implementation utilized function-merging and block-reuse techniques to distinguish between ventricular and non-ventricular ectopic beats with a dynamic power of 26 μW [46]. A quantized MLP combined with bCNN was introduced for binary classification and demonstrated an accuracy of 98.5% [47]. Nevertheless, owing to the extremely low bit quantization, most previous bCNN works have only focused on the binary classification of ECG signals and it is still a challenge to realize multi-classification using bCNNs.

To compensate for the accuracy loss of adopting bCNNs for multi-classification, a higher input data resolution and more nodes per layer, as well as more layers, are required. However, in order to achieve reasonable accuracy, augmented networks are often noticeably more complex than the original bCNN. As a speedup strategy, depthwise separable convolution (DSC), which breaks a conventional convolution layer into a depthwise (DW) convolution plus a 1 × 1 pointwise (PW) convolution, has been extensively used in lightweight CNNs and has been proven to be able to reduce computational resources significantly [49,50,51,52,53]. A DSC layer was first employed in MobileNet [49] to cut down both the model size and the number of operations. Then, DSC combined with a CNN was utilized in ECG classification [52,53] and achieved a noticeable reduction in the number of convolutional parameters. In addition, DSC was also applied in bCNNs [54,55] to decrease the computational complexity of bCNNs for keyword spotting multi-classification tasks. Thus, for the multi-classification of ECG signals using bCNN, DSC provides a viable approach to reduce the complexity induced by the accuracy compensation network discussed above.

**Figure 1:**
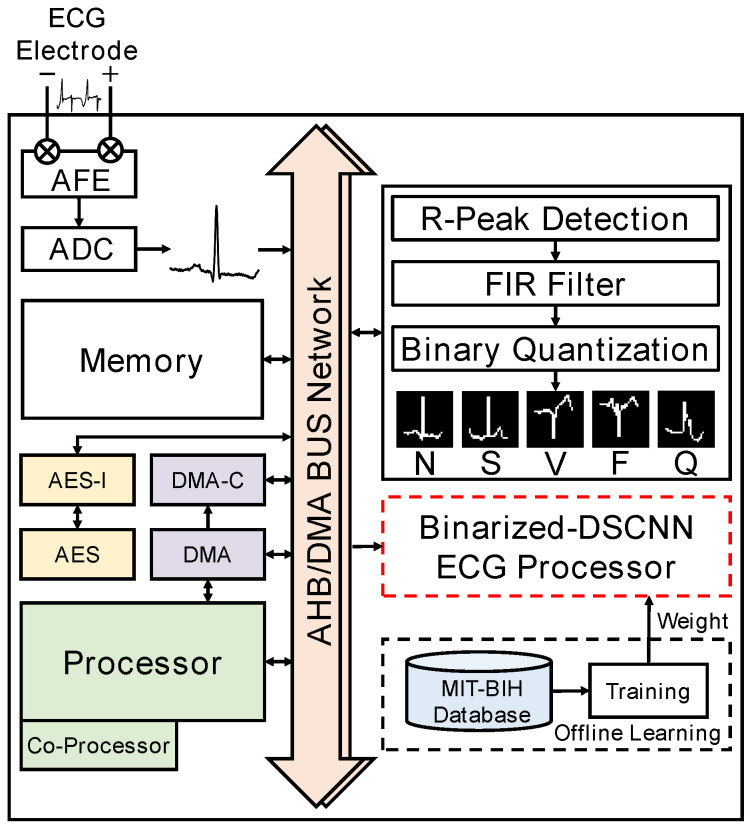
The SoC architecture with the bDSCNN model.

Adopting a general system-on-a-chip (SoC) architecture [56,57,58], as shown in Figure 1, this work proposes a five-type ECG signal classifier utilizing a binarized depthwise separable convolutional neural network (bDSCNN). While traditional CNNs operate convolution and pooling sequentially, the proposed method adopts a merged convolution–pooling (MCP) layer that combines the convolution and pooling layers to reduce the number of operations. Moreover, since the $\left\{0,1\right\}$ binarization method is utilized, the binarized weights and activation coefficients allow the multiplication to be simplified as AND logic, reducing the hardware resources required for multi-classification.

Although this paper is only focused on bDSCNN, a complete ECG monitoring system also requires an analog front-end (AFE) for tasks such as signal amplification, DC blocking, anti-aliasing filtering, dynamic range alignment, and signal digitization using an analog-to-digital converter (ADC) [36,46]. Noise control techniques are also needed to remove various artifacts such as loose lead artifacts, muscle tremor artifacts, etc.

In summary, this paper proposes a bDSCNN model for multi-class ECG signal classification implemented in an FPGA platform, with the following features:

1. A bDSCNN model based on the $\left\{0,1\right\}$ binarization approach and a binarized DSC (bDSC) layer with optimized hardware resource consumption are adopted. Therefore, the number of required parameters and computations are decreased compared with a bCNN model based on $\left\{-1,1\right\}$ binarization.
2. An MCP method is proposed to eliminate the repetitive computations and achieves an efficient hardware implementation. It does not introduce any accuracy loss compared with the traditional processing method.
3. A blockwise incremental calculation is designed to reduce computations and redundant repetitive storage compared with the traditional computation strategy.
4. R peak interval data and P-QRS-T features are fed into the bDSCNN model to improve the classification accuracy.

The rest of this paper is organized as follows. Section 2 gives the methods, including model design and hardware design of the bDSCNN model for multi-class ECG signal classification. The results are listed in Section 3. The discussions are listed in Section 4. Finally, Section 5 concludes this paper.

## 2. Methods

### 2.1. Model Design

The proposed bDSCNN model for multi-class ECG signal classification is first designed and trained in a software environment and then implemented on an FPGA platform. This section focuses on the software-based model design process.

#### 2.1.1. Basic Model Structure

**Figure 2:**
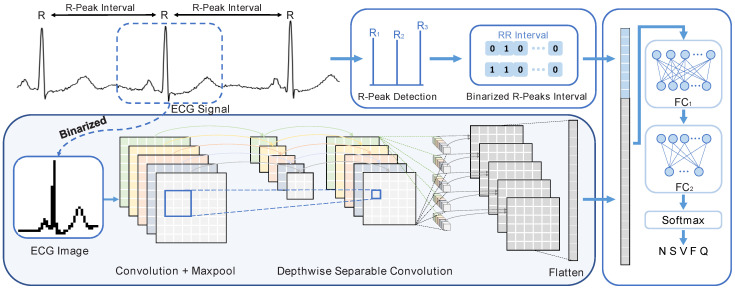
Overall design of the bDSCNN model.

The structure of the bDSCNN model is shown in Figure 2. The inputs of the model include 2D ECG images and extracted R peak interval data, which are combined together to improve the classification accuracy. The P-QRS-T features are extracted from 2D ECG images by using convolution and max-pooling layers, as well as a DSC layer. The R peak interval data represent the interval of the ECG signal between two adjacent R peaks (RR) [36]. Finally, these features are fed into the fully connected (FC) layers, followed by a five-category softmax output layer. Taking the convenience of hardware implementation into account, the proposed bDSCNN model uses the $\left\{0,1\right\}$ binarization method, formulated as

(1) $$Binarized\left(x\right)=\left\{\begin{array}{cc} 1, & \;if\;x>0\\ 0, & \;otherwise \end{array}\right.,$$

where *x* represents the weights and output values of each layer.

Features of the binarized ECG image are extracted by conventional convolution with multiple convolution kernels whose kernel size is 3 × 3 and then suppressed by the max-pooling operation. As a result, a single-channel input image is transformed into multiple-channel feature maps. As described in Section 1, to maintain the accuracy of multiple classifications for ECG signals in the bCNN approach, an image with a higher resolution is required. The additional number of parameters introduced by high-resolution images must be handled using a more complex model. To decrease the model complexity, a bDSC layer is used to deconstruct the conventional 3D convolution into a 2D DW convolution plus a PW convolution. Using *N* convolution kernels with a kernel size of 3 × 3 for each channel, a traditional convolution operation is used to yield the number of parameters of (9 × N) (i.e., (3 × 3) × *N*), while the parameter number of DSC drops to (9+N) (i.e., (3 × 3)+(1 × 1) × *N*). If N=18, a parameter number reduction of six times can be achieved.

In addition, the batch normalization (BN) layer has been proven to be crucial for the successful training of bCNN networks, and it can guarantee stable training with a higher learning rate and model accuracy as well as faster training speed [59,60,61]. Therefore, BN layers are inserted after the DSC and the FC layers. The value *x* is normalized with the BN layer as

(2) $$\begin{array}{c} \hat{x}=\frac{\gamma (x-\mu )}{\sqrt{\sigma^{2}+\epsilon }}+\beta , \end{array}$$

where $\hat{x}$ represents the normalized value of *x* after the BN layer. γ and β represent the scaling and translation parameters that need to be learned in the BN layer, respectively. μ and σ represent the mean and standard deviation, and ϵ is a parameter to prevent the denominator from being 0.

#### 2.1.2. Database and Software Configuration

The MIT-BIH Arrhythmia Database, developed by the Massachusetts Institute of Technology and Beth Israel Hospital, is a widely utilized repository containing 48 digitized electrocardiogram signals of two-channel ambulatory ECG recordings obtained from 47 subjects [62]. These recordings were acquired at a 360 Hz sampling frequency with 11-bit amplitude resolution. This database is used to assess the performances of the proposed model.

**Figure 3:**
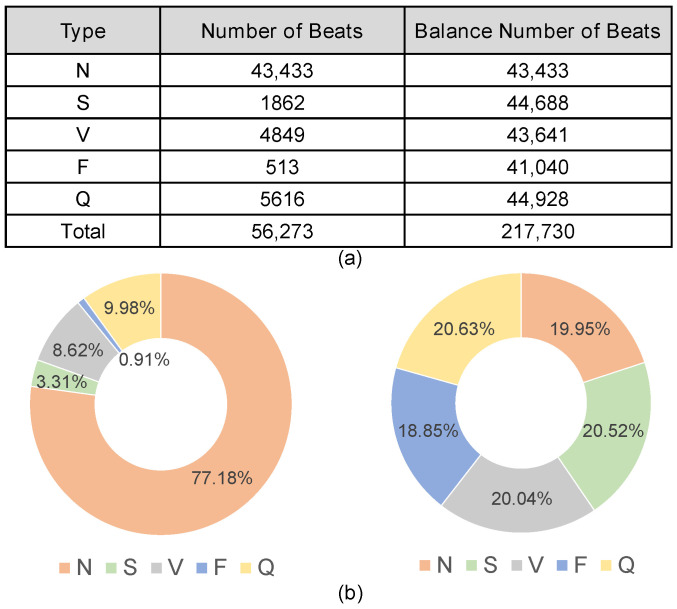
(**a**) The numbers of the five original classes and balanced beat subtype for training. (**b**) The proportions of the original and balanced data for the five types of beat data in training.

According to the protocols established by the Association for the Advancement of Medical Instrumentation (AAMI) [63], non-life-threatening arrhythmias can be divided into five main categories: non-ectopic (N), supraventricular ectopic (S), ventricular ectopic (V), fusion (F), and unknown (Q). In this work, a conditional data grouping scheme [36] is employed to guarantee sufficient samples in training. For each patient record, 70% of the data are randomly selected as training data, and the remaining 30% are further divided into 30% validation data and 70% testing data to continuously monitor the loss of the bDSCNN model during the training process. Considering the imbalance of the ECG signal classes for training, various data augmentation schemes have been proposed to balance the dataset [64,65,66,67,68]. In this work, the Z-score data augmentation method is used to generate the non-N-type heartbeat data by varying the mean and standard deviation of the Z-score calculated from the original ECG signals. After dataset expansion, the total number of heartbeats for training, including N, S, V, F, and Q classes, increases from 56,273 to 217,730, as shown in Figure 3. The maximum proportion of the heartbeat number to the total heartbeats decreases from 77.18% to 19.95%, while the minimum proportion increases from 0.91% to 18.85%. Consequently, the number of samples for each class becomes more balanced and is more suitable for model training. For validation and testing data, data augmentation is not performed, and the testing data numbers of N, S, V, F, and Q are 13,001, 583, 1478, 157, and 1696, respectively.

**Figure 4:**
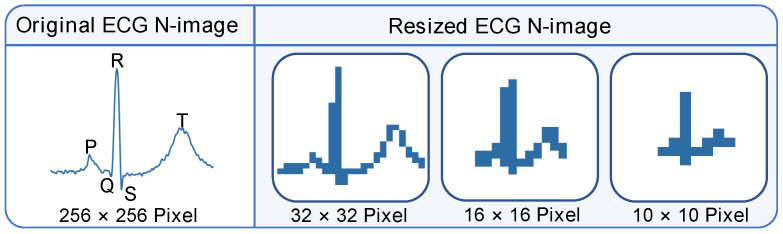
The original ECG N-image and the resized ECG N-image.

BCNNs require the transformation of 1D ECG signals into 2D images for capturing spatial structural features. Given the MIT-BIH dataset has R peaks annotated for each ECG beat, 300 ECG samples are taken around the R peaks (100 and 200 samples on the left and right of the R peaks), and the R peak interval data are calculated by measuring the time between consecutive R peaks. The Python 3 programming language and open-source OpenCV2 library are then employed to transform the samples into an image. The original and resized ECG images are shown in Figure 4. Larger sizes of ECG images offer more detail, but require more complex model structures and additional hardware resources. Smaller-size images lead to simpler model structures and less hardware resources, but suffer from less distinct P-QRS-T features. Taking both the classification accuracy and the model complexity into consideration, a binarized ECG image size of 32 × 32 px is chosen.

The proposed bDSCNN model is trained using Python 3.8 with the Keras library on a 3.20 GHz AMD Ryzen 7 with Nvidia RTX 2050 GPU. The Adam optimizer is chosen, with starting and ending learning rates of $10^{-3}$ and $10^{-4}$, respectively. To automatically determine the epoch size, early stopping techniques are implemented to ensure that the model does not overfit.

### 2.2. Hardware Design

Using the model structure described in Section 2.1.1, all the layers of the bDSCNN model are implemented in hardware. In addition to the bDSC method, two other mechanisms are adopted to reduce the usage of hardware resources. First, instead of conducting convolution and max-pooling operations sequentially, an MCP method that merges the pooling with the convolution layer is proposed to save the number of operations. Second, a blockwise incremental calculation is designed by reconstructing the computation process of feature extraction to reduce computation operations as well as memory access.

#### 2.2.1. MCP Layer Implementation

**Figure 5:**
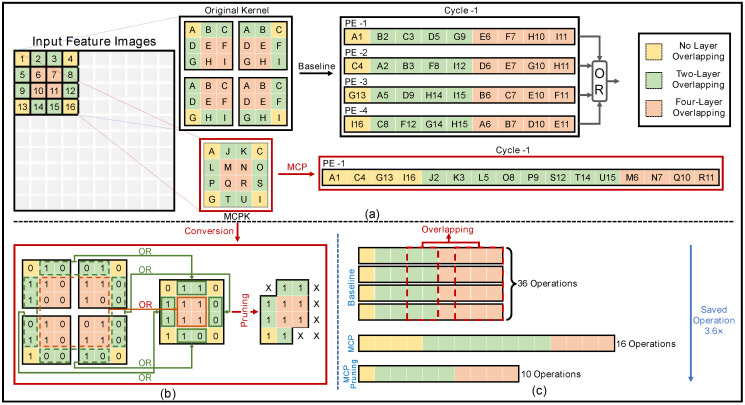
The proposed MCP method with kernel size of 4 × 4 and transposed stride of 2: (**a**) the comparison of parallel computations between “baseline” and MCP methods; (**b**) the reconstruction process of the proposed merged convolution–pooling kernel; (**c**) the comparisons of operation numbers between the “baseline” and MCP methods.

The MCP method and a comparison of it with the traditional sequential convolution and pooling method are illustrated in Figure 5. As shown in Figure 5a, the traditional “baseline” bCNN convolves the image using four identical filters with a kernel size of 3 × 3 and a transposed stride of 1. The elements of the convolution kernel are labeled with letters A–I. As a result, an input 4 × 4 px matrix whose elements are labeled with numbers 1–16 is converted to a 2 × 2 output feature matrix after the convolution operation. The subsequent max-pooling down-samples the feature matrix to 1 px by a 2 × 2 pooling kernel. The traditional convolution contains many repetitive operations, as highlighted by three different colors. To achieve higher efficiency, those repetitive operations can be saved.

This work proposes an MCP method that combines convolution and pooling operations to solve the above problem. The key idea is to reconstruct an equivalent 4 × 4 convolution kernel by merging the original four 3 × 3 kernels. The kernel values of yellow regions that are not overlapping in traditional convolution are retained. The two neighboring 1 × 2 or 2 × 1 green overlapping regions are merged using OR operations; for example, J = B|A, K = C|B. The four 2 × 2 red overlapping regions in traditional kernels are merged using OR operations as well; for example, M = E|D|B|A. Consequently, the repetitive convolution operations in traditional convolution are combined, and the number of operations is reduced. With the reconstructed kernel, the max-pooling layer in the traditional CNN is integrated with the convolutional layer via OR operations, and a following pooling layer is no longer needed. Because the new convolution is equivalent to the traditional one from the output perspective, the MCP method achieves the same accuracy using noticeably fewer operations.

The merged convolution–pooling kernel (MCPK) is constructed as the last step of the training process, and its calculation procedure is given in Algorithm 1. The algorithm checks the element position of the original convolution kernel, performs OR computations for the overlapping regions, and preserves the values for the nonoverlapping regions. If the weight after transformation is zero, its corresponding branch is pruned. Figure 5b gives an example to further illustrate the above procedure.

**Figure 6:**
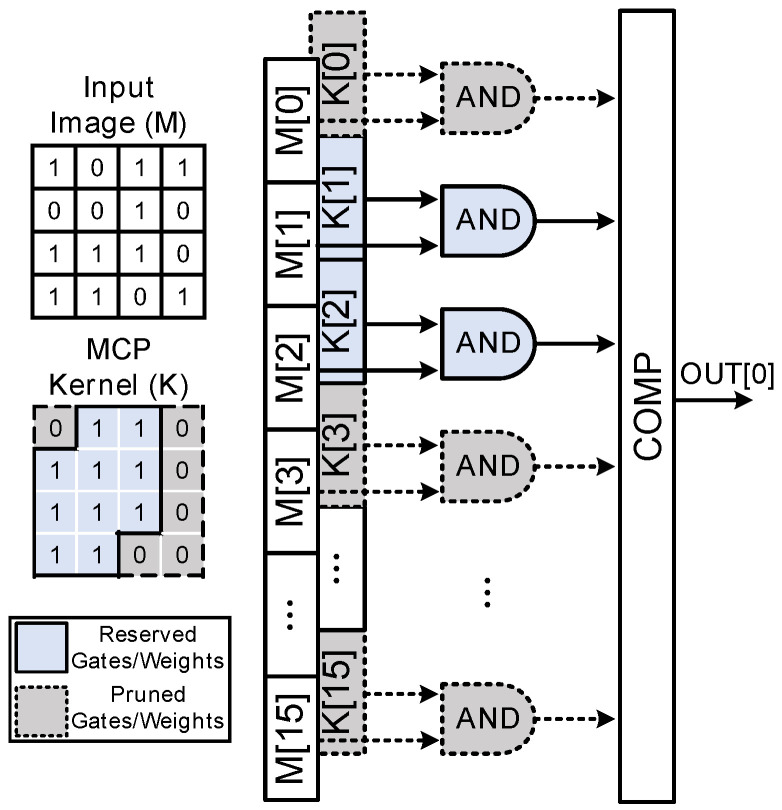
Illustration of the pruning process in the MCP method.

Figure 5c compares the number of operations between the proposed MCP and the “baseline” methods. One PE in the traditional methods consists of nine AND operations to realize a convolution operation, and 36 AND operations in total are required to perform four convolution operations. In contrast, in the proposed MCP method, only one PE, which contains 16 AND operations, is required. With an MCP kernel as shown in Figure 5b, the AND operations with “0” as input can be reduced. Figure 6 further provides the detailed hardware implementation for the pruning process. As a result, 16 AND operations are reduced to 10 operations, which is 3.6 times less compared with the traditional method.

**Algorithm 1: **MCPK Weight Calculation

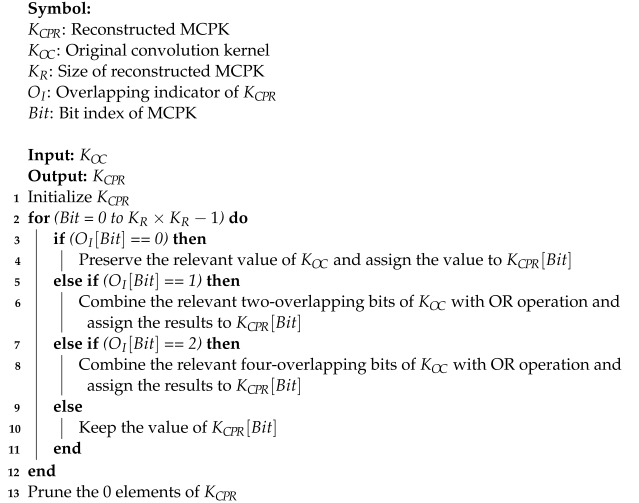



Note that the proposed MCP method is not limited to convolution kernels with 3 × 3 size; it can also be applied to kernels with other sizes. The size of the reconstructed MCPK $K_{R}$ and the stride size $S_{R}$ can be expressed as

(3) $$\begin{array}{c} K_{R}=K_{O}+\left(S_{O}\times \left(P-1\right)\right), \end{array}$$

(4) $$\begin{array}{c} S_{R}=S_{O}\times S_{P}, \end{array}$$

where $K_{O}$, $S_{O}$, *P*, and $S_{P}$ represent the original convolution kernel size, original convolution stride, pooling size, and original pooling stride, respectively.

#### 2.2.2. Blockwise Incremental Calculation

**Figure 7:**
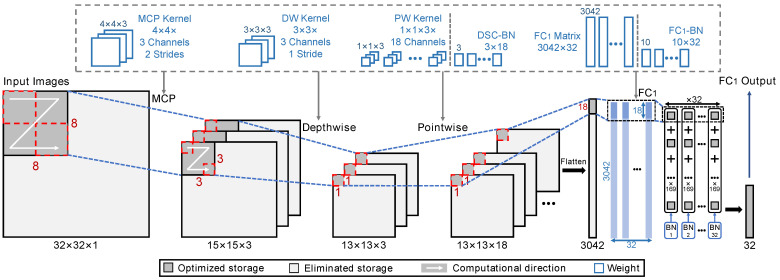
Blockwise incremental calculation to eliminate repetitive storage and computations in the bDSCNN.

To reduce computations and eliminate repetitive storage between the input image and the first FC (${\mathrm{FC}}_{1}$) layer, a blockwise incremental calculation scheme is adopted in the hardware implementation. The blockwise incremental computation method optimizes the inference process of the model without changing the parameters and structure, thus maintaining the same classification accuracy. Different from the traditional layer-by-layer calculation, in the blockwise incremental calculation process, the input images are reorganized into multiple blocks, and the blocks are processed one at a time through MCP, DSC-DW, DSC-PW, and ${\mathrm{FC}}_{1}$ layers until all the blocks are traversed and ${\mathrm{FC}}_{1}$ results are derived. The data flow of the blockwise incremental calculation is shown in Figure 7. The input image is convolved by multiple 4 × 4 MCPKs to form multichannel output feature maps. Then, the DSC operation is performed with three 3 × 3 DW convolution kernels and eighteen 1 × 1 × 3 PW convolution kernels to derive a 1 × 1 × 18 DSC feature map. In the following ${\mathrm{FC}}_{1}$ layer, the feature map is converted to 32 FC_1_ intermediate results by an 18 × 32 FC_1_ conversion matrix. To cover the full input image, the above operations are repeated 169 (13 × 13) times, and the results from each iteration are combined and normalized to derive the final ${\mathrm{FC}}_{1}$ output result.

The saving of memory by adopting the blockwise incremental calculation can also be seen from Figure 7, in which the dark gray regions represent optimized storage for the process of feature extraction, and the light gray regions represent the eliminated data storage. Instead of storing all of the intermediate features, whose sizes are 32 × 32, 15 × 15 × 3, 13 × 13 × 3, 13 × 13 × 18, and 3,042, for each operation in the traditional layer-by-layer method, the blockwise incremental calculation only requires 8 × 8, 3 × 3 × 3, 1 × 1 × 3, 1 × 1 × 18, and 18 data blocks to store the intermediate features.

**Figure 8:**
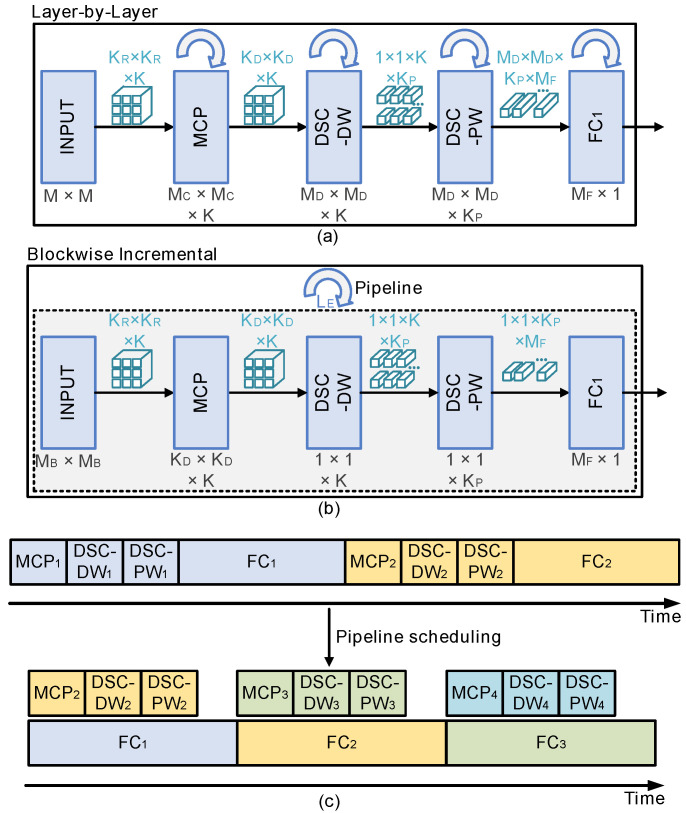
(**a**) The latency of traditional layer-by-layer calculation. (**b**) The latency of blockwise incremental calculation. (**c**) Pipeline scheduling for blockwise incremental calculation.

Figure 8a,b show the latency of the traditional layer-by-layer calculation and the blockwise incremental calculation. In the traditional calculation, each layer of the model is computed independently, and the latency is the sum of the computation time for each layer. For example, the ‘MCP’ layer performs calculations $M_{C}\times M_{C}$ times using the $K_{R}\times K_{R}\times K$ MCP kernels. The ‘DSC-DW’ and ‘DSC-PW’ layers perform calculations $M_{D}\times M_{D}$ times using $K_{D}\times K_{D}\times K$ DW kernels and $K\times K_{P}$ PW kernels. In the ${\mathrm{‘FC}}_{1}$’ layer, the ‘DSC-PW’ results are calculated $M_{D}\times M_{D}\times K_{P}$ times by using an $M_{F}\times 1$ matrix. In this work, the size of the feature map can be calculated by

(5) $$\begin{array}{c} M_{C}=\frac{1}{2}\times \left(M-K_{R}+P\right), \end{array}$$

(6) $$\begin{array}{c} M_{D}=M_{C}-K_{D}+1, \end{array}$$

where *M*, $M_{C}$, $M_{D}$, and $K_{D}$ represent the ECG image size, the MCP feature map size, the DSC feature map size, and the DSC kernel size, respectively. For the traditional calculation method, the latency (*L*) can be calculated by

(7) $$\begin{array}{c} L=M_{C}^{2}+2\times M_{D}^{2}+K_{P}\times M_{D}^{2}, \end{array}$$

where $K_{P}$ represents the number of DSC-PW kernel channels.

In the blockwise incremental calculation, the ‘MCP’, ‘DSC-DW’,‘DSC-PW’, and ${\mathrm{‘FC}}_{1}$’ blocks are executed sequentially, and the latency is the multiplication results of the computation time for each block and the number of repetitions of each cycle. For each computation of the blockwise incremental calculation, the ‘MCP’ block performs calculations for $K_{D}\times K_{D}$ times using the same MCPKs.

The ‘DSC-DW’ and ‘DSC-PW’ blocks perform calculations once using the same DW/PW kernels. As for the ${\mathrm{‘FC}}_{1}$’ block, the DSC-PW results are calculated $K_{P}$ times by using the same matrix. Thus, the latency of the blockwise incremental calculation ($L_{B}$) can be calculated by

(8) $$\begin{array}{c} L_{B}=\left(K_{D}^{2}+1+1+K_{P}\right)\times M_{D}^{2}. \end{array}$$

For the model used in this work, the number of latencies *L* and $L_{B}$ are calculated to be 3605 and 4901, respectively. To reduce the extra latency introduced by blockwise incremental calculation, a pipeline scheduling scheme is proposed as shown in Figure 8c, where the ‘MCP,’ ‘DSC-DW,’ and ‘DSC-PW’ processes for the next block of an input image are scheduled in parallel with the current ${\mathrm{‘FC}}_{1}$’ process. Before adopting the pipeline scheduling, the time consumption of one single pipeline stage is the sum of the ‘MCP,’ ‘DSC-DW,’ ‘DSC-PW,’ and ${\mathrm{‘FC}}_{1}$’ process times. After re-scheduling, the latency becomes only the sum of the ${\mathrm{‘FC}}_{1}$’ process time and the latency $L_{B}$ can be written as

(9) $$\begin{array}{c} L_{B}=\left(K_{P}\right)\times M_{D}^{2}. \end{array}$$

Therefore, the latency of the blockwise incremental calculation is reduced to 3042 by using the pipeline scheduling, which is even less than the layer-by-layer latency. Note that the same three MCPK configurations for both layer-by-layer and blockwise implementations are assumed when deriving the above data; latency performances could be further improved by employing more MCPKs for both implementations.

#### 2.2.3. Batch Normalization

As described in Section 2.1.1, BN allows stable training at larger learning rates to improve training speed and training accuracy. BN layers are inserted after the DSC, ${\mathrm{FC}}_{1}$, and second FC (${\mathrm{FC}}_{2}$) layers to achieve better performance in model training. After the DSC and ${\mathrm{FC}}_{1}$ layers, the BN layer is followed by a binarized activation layer. The BN transformation in the hardware can be simplified by combining the BN layer and the activation layer. Referring to (1) and (2), the combined activation function can be written as

(10) $$\begin{array}{c} Binarized-BN\left(x\right)=\left\{\begin{array}{cc} 1, & \;if\;x>\lfloor \mu -\frac{\beta \sqrt{\sigma^{2}+\epsilon }}{\gamma }\rfloor \\ 0, & \;otherwise \end{array}\right.. \end{array}$$

Since the direct calculation of $\mu -\frac{\beta \sqrt{\sigma^{2}+\epsilon }}{\gamma }$ demands high hardware resource usage, the threshold of the function Binarized-BN(x) is calculated in software according to (10) and then stored in the BRAM block. For example, assuming that μ=0.8035, β=−2.0248, σ=0.9242, γ=0.7093, and ϵ=0.0001, the BN layer threshold is calculated to be 3.4418 and rounded down to 3. The binarized-BN operation is then performed through a comparator in the hardware using the transformed threshold. As for the ${\mathrm{FC}}_{2}$-BN layer, the output of the ${\mathrm{FC}}_{2}$ is 5 × 5-bits, resulting in 32 possible values for each result. For the purposes of efficient hardware implementation, the ${\mathrm{FC}}_{2}$-BN layer is realized as a lookup table whose entries are calculated by referring to (2) in the software.

#### 2.2.4. Hardware Architecture

**Figure 9:**
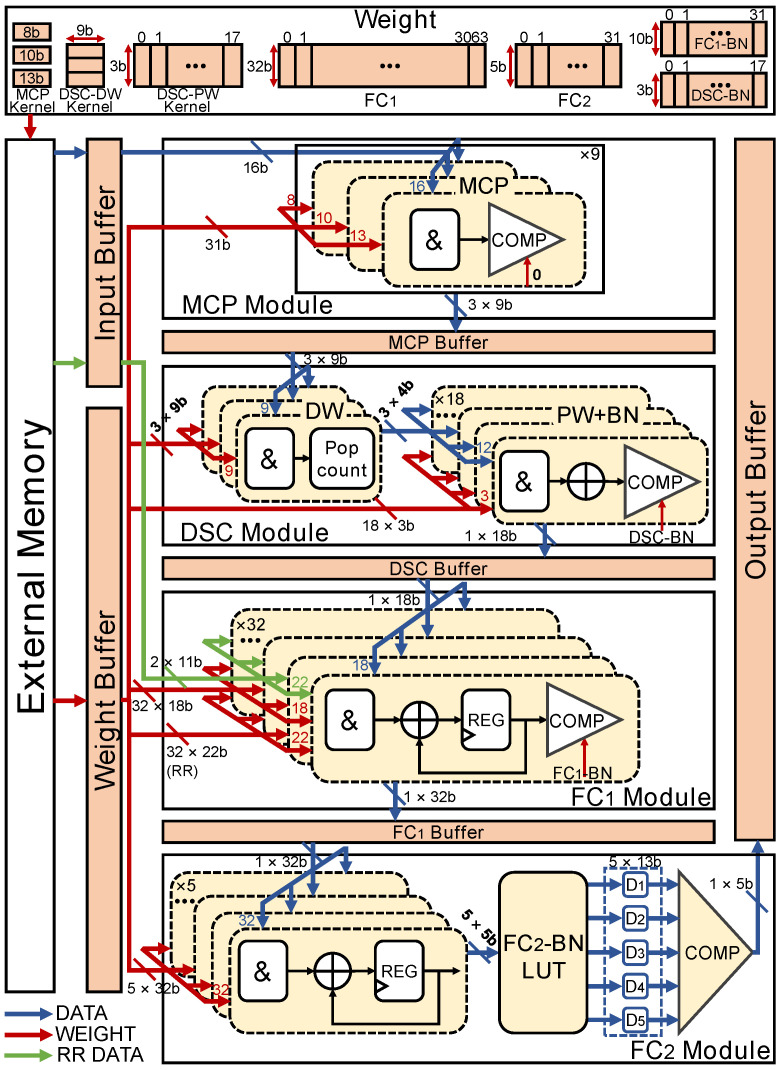
The overall hardware architecture of the proposed bDSCNN.

The overall hardware architecture of the proposed bDSCNN inference accelerator is shown in Figure 9. The weights of the proposed model are stored in the external memory and can be loaded for classification computation via a weight buffer. In addition to the MCP, DSC, and two FC modules, the system also employs an input buffer to store the input image and the RR interval data, storage buffers for the MCP, DSC, and ${\mathrm{FC}}_{1}$ layers, and an output buffer to store the classification result. The MCP module contains three kernels and each kernel is calculated with the input block image. The computation of each kernel is performed using AND gates and a comparator. To derive a 3 × 3 MCP feature map as shown in Figure 7, the above computation needs to be repeated nine times for one MCPK.

The DSC module consists of three DW convolutions and 18 PW convolutions. Each DW convolution individually convolves with the corresponding channel of the feature maps. Each DW convolution also has its own 3 × 3 DW kernel and is implemented with nine two-input AND gates and a population count (popcount) unit. The PW convolution is performed by three 4-bit AND gates, one accumulator, and one comparator for each output channel. The threshold of the comparator is a normalized value transformed by the BN activation function.

The ${\mathrm{FC}}_{1}$ module consists of 32 blocks, with each block containing a two-input AND gate, an accumulator, a register, and a comparator. For each block in the ${\mathrm{FC}}_{1}$ module, to save the hardware resources, the multiplication of the ${\mathrm{FC}}_{1}$ input vector and the weight vector is calculated in multiple cycles, and the result of each cycle is accumulated. Then, the 32 12-bit results are compared with ${\mathrm{FC}}_{1}$-BN thresholds to obtain 32 1-bit ${\mathrm{FC}}_{1}$ outputs. For the ${\mathrm{FC}}_{2}$ module, the multiplication of the 32-bit ${\mathrm{FC}}_{1}$ output and the five 32-bit ${\mathrm{FC}}_{2}$ weight vectors yields five 5-bit output results. Then, the five results are fed into the ${\mathrm{FC}}_{2}$-BN lookup table to obtain five 13-bit ${\mathrm{FC}}_{2}$-BN layer output results. Finally, the BN results are compared to obtain the classification result in one-hot format.

## 3. Results

Based on the structure of the proposed bDSCNN, the model configurations for both the software and hardware implementations are listed in Table 1. This section provides the experimental results and comparisons with state-of-the-art works.

### 3.1. *Model Performance*

To verify the effectiveness of the proposed bDSCNN, several models with different structures are trained, tested, and evaluated by standard metrics including loss, $C_{operation}$, accuracy (Acc), and macro-F1, where loss represents the value calculated by the loss function. In this work, the loss function is selected as the cross-entropy function. $C_{operation}$ represents the number of the convolutional operations of the model. Acc and macro-F1 are defined as

(11) $$\begin{array}{c} Acc=\frac{TP+TN}{TP+TN+FP+FN}, \end{array}$$

(12) $$\begin{array}{c} Macro-F1=\frac{1}{N}\sum_{i=1}^{N}F1-score_{i}, \end{array}$$

where TP, TN, FP, and FN denote true positive, true negative, false positive, and false negative, respectively. *N* represents the number of ECG signal classes. For the five-classifier in this work, *N* is 5. The F1-score is a standard metric for two classifiers, which is described as

(13) $$\begin{array}{c} F1-score=\frac{2\times TP}{2\times TP+(FP+FN)}. \end{array}$$

In the experiments, the number of channels is selected through an incremental search based on the classification accuracy and the macro-F1 score. As the number of channels increases, the accuracy also increases, until it reaches the maximum value, then it declines. The number of channels at the maximum point is considered to be the optimal choice. As a result, 3 and 18 are selected as the numbers of channels for the first convolution layer and the DSC layer, respectively. Table 2 lists the performance comparisons of various bCNN structures. In Table 2, “NoBN” refers to being without the BN layer. “SC” denotes that the second convolution layer uses traditional convolution. As Table 2 shows, the proposed bDSCNN model with the concat RR interval, the BN layer, and the second DSC convolution layer demonstrates improved performance, achieving a testing loss of 0.1099, 13,689 convolutional operations, an accuracy of 96.61%, and a macro-F1 score of 89.08%.

The model with the overall best performance is stored and executed on an SoC device for inference. Intel’s Cyclone V-based DE1-SoC is chosen as the target FPGA platform, and the weights of the well-trained bDSCNN are stored in its internal BRAM for deployment.

### 3.2. *Algorithm Accuracy*

The testing results of the proposed bDSCNN network are shown in Table 3. The statistics listed in the confusion matrix are the predicted numbers of corresponding ECG signals. In addition, two-class accuracy, five-class accuracy, macro-F1, sensitivity (Sen), positive predictive value (Ppv), and specificity (Spec) are also employed to evaluate the performance of the model.

These criteria are defined as follows:

(14) $$\begin{array}{c} Sen=\frac{TP}{TP+FN}, \end{array}$$

(15) $$\begin{array}{c} Ppv=\frac{TP}{TP+FP}, \end{array}$$

(16) $$\begin{array}{c} Spec=\frac{TN}{TN+FP}. \end{array}$$

As shown in Table 3, the proposed model has a five-class accuracy of 96.61% and a macro-F1 score of 89.08%. Meanwhile, a maximum F1-score of 98.02% with corresponding two-class accuracy of 96.96%, a sensitivity of 97.82%, a positive predictive value of 98.21%, and a specification of 94.07% are achieved.

### 3.3. *Model Complexity and Hardware Resource Usage*

The complexity and performance of the bDSCNN model are compared with those of other reported ECG classification works employing CNN methods in Table 4. As the table shows, for ECG classifiers that adopt a 1D-CNN with multi-bit input data, more convolution layers and kernels are necessary to achieve high accuracy. In terms of model complexity, the bDSCNN model uses only 2 convolution layers and 24 convolution kernels. The numbers of total kernel parameters and multiply–accumulates (MACs) are reduced to 108 and 137,547, respectively. By adopting the MCP method, the number of convolution–pooling operations is reduced from 25,650 to 10,800 in the bDSCNN model, resulting in a total operation number of 122,697, which is the minimum among similar works. As for bCNN, this work achieves an increased number of classifications at the cost of higher input data resolution and more layers. Although the weights and activation values of the proposed model are compressed to 1 bit for less complexity, the classification accuracy is comparable to that of other multi-classifiers using multi-bit CNN models.

The performances of several hardware implementations are summarized in Table 5. As shown in the table, although the MLP approaches show relatively low hardware usage, they require additional extractors to extract features that are necessary for successful classification metrics. At the same time, it can be seen that the bCNN classifiers demand the least hardware resources owing to the binarization of their internal weights and activation values.

To reduce the extra storage caused by higher image resolution for five-type ECG signal classification, the blockwise incremental calculation method is employed. This results in a 90% reduction in the storage of feature maps, from 5285 registers to 542 registers, compared with the traditional layer-by-layer calculation. Thus, the number of LUTs and REGs (hardware resources) used in this work is 3799, less than those used in previous works. The number of DSP blocks used is 0 in this work because the multiplication and addition operations inside the bCNN are simplified to AND operations.

In addition, since most binary ECG classifiers mainly distinguish between V and non-V signals, the comparison metrics for binary classifications are also listed in Table 5. In this work, the classification accuracy and F1-score are approximately 98.7% and 92.9%, respectively. The number of clock cycles per classification is 3087. Using the Altera Powerplay Power Analysis tool, the dynamic power and energy per classification are evaluated as 20 μW and 617.4 nJ, respectively, when operating at a 100 KHz clock frequency.

## 4. Discussion

### 4.1. Conversion of 1D Signals to 2D Images

In this work, the proposed 2D bDSCNN model is used to classify the ECG signal, which requires the conversion of 1D ECG signals to 2D images. The reason for the conversion is that the 2D images can provide additional spatial dimension information compared with the 1D ECG signal. Meanwhile, processing the 2D images allows for extreme quantization to 1-bit data width compared with the multi-bit data widths required for processing 1D ECG signals. This enables the complex multiplication calculations of 1D signals to be simplified to AND gate operations of 2D images, reducing overall hardware resource consumption.

Although full-bit map image conversion preserves all spatial information, it demands substantial hardware resources for processing. To reduce hardware resource consumption, image compression can be employed at the cost of an acceptable classification accuracy loss. In this work, with both hardware resource consumption and classification accuracy in consideration, a 32 × 32 px image size is selected to achieve a balanced performance between model accuracy and hardware complexity.

### 4.2. Dataset Splitting Methods

This work splits the MIT-BIH dataset using the patient-specific method, as most previous hardware-related works did [25,36,46,47,65,70], for fair comparison purposes. However, in real applications involving new patients, the patient-wise dataset-splitting method is also frequently used. To further validate the proposed method, a separate model using the patient-wise splitting scheme [67,68,71] is trained and compared.

As shown in Table 6, the bDSCNN model trained using the patient-specific dataset-splitting method has a higher accuracy compared with the model trained using the patient-wise method. At the same time, the model based on the patient-wise dataset needs additional convolution kernels to extract features, leading to more complex topologies and parameters, and thus, more hardware resources and higher classification latency than the model based on the patient-specific dataset. This performance change might be caused by the fact that the ECG morphologies are often different among patients, which in turn leads to a bigger difference between the testing and the training data for the model trained by the patient-wise dataset. This problem could potentially be solved by the on-chip learning method, which can fine-tune the model on the fly to adapt to each of the testing patients [71].

## 5. Conclusions

In this paper, an efficient bDSCNN model was proposed and implemented for the classification of multi-class ECG signals. The proposed model adopted $\left\{0,1\right\}$ binarization method for the convenience of hardware implementation. The MCP method was designed to achieve the fusion of convolution and pooling operations by reconstructing the MCPK to reduce the repetitive computations in traditional CNN methods. Meanwhile, a blockwise incremental calculation was adopted to eliminate redundant storage and computations. The proposed bDSCNN model was evaluated on an Intel DE1-SoC FPGA and achieved comparable classification accuracy with less model complexity compared to other multi-class ECG signal classifiers based on FPGA. The proposed bDSCNN model achieves a five-class classification of 96.61% and a macro-F1 score of 89.08%, with 3.8k LUTs plus REGs and dynamic power dissipation of 20 μW.

## Figures and Tables

**Table 1:** Model configurations in software and hardware.

Size	Software			Hardware			
	Layer	Weight	Output Format	Layer	Weight	Output Format	Result-Reg (Bits)
32 × 32	Input	-	(32, 32, 1)	Input	-	(8, 8, 1)	64
	Conv-Valid Binarized Max-Pooling	3 × 3 × 3 - -	(30, 30, 3) - (15, 15, 3)	MCP	8 + 10 + 13	(3, 3, 3)	27 *
	DSC-DW	3 × 3 × 3	(13, 13, 3)	DSC-DW	3 × 3 × 3	(1, 1, 3)	12 * (3 × 4-bit)
	DSC-PW BN-DSC Binarized	1 × 1 × 3 × 18 18 × 4	(13 ,13 ,18 ) - -	DSC-PW	126	(1, 1, 18)	18 *
	FC_1_ BN_1_ Binarized	13 × 13 × 18 × 32 32 × 4 -	32 - -	FC_1_	97,472	32	416 (32 × 12-bit + 32-bit)
	FC_2_ BN_2_ Softmax	32 × 5 5 × 4 -	5 - 5	FC_2_	180	5	5

* Omitted due to blockwise incremental calculation.

**Table 2:** The performance enhancements of different model structures for offline learning.

Method		Acc (%)	Macro-F1 (%)	Loss	C_*operation*_
No RR	bDSCNN-NoBN	77.28	57.40	1.1677	13,689
interval	bCNN-SC	96.05	86.07	0.1425	82,134
concat	bDSCNN	95.87	85.96	0.1474	13,689
RR	bDSCNN-NoBN	79.97	60.34	1.0521	13,689
interval	bCNN-SC	96.66	89.15	0.1096	82,134
concat	bDSCNN	96.61	89.08	0.1099	13,689

**Table 3:** Confusion matrix and evaluation metrics for ECG heartbeats.

		Original					Sen (%)	Ppv (%)	Spec (%)	Two-Class Acc (%)	Five-Class Acc (%)	Macro -F1 (%)
		N	S	V	F	Q						
	N	12,718	98	57	20	57	97.82	98.21	94.07	96.96		
	S	134	469	3	0	2	80.45	77.14	99.15	98.50		
Predicted	V	102	15	1410	12	18	95.40	90.56	99.05	98.73	96.61	89.08
	F	29	1	6	125	0	79.62	77.64	99.79	99.60		
	Q	18	0	2	0	1619	95.46	98.78	99.87	99.43		

**Table 4:** Model complexity and performance evaluation.

	TCAS-I 2022 [65]	TBioCAS 2019 [25]	IRBM 2022 [69]	TBioCAS-BP ^1^ 2021 [46]	This Work
Convolution Type	1D	1D	2D	2D	2D
Input data Resolution	16-bit	11-bit	8-bit	1-bit	1-bit
No. of Input Samples	320	400	64 × 64	16 × 20	32 × 32
No. of Kernels	120	48	170	16	24
No. of Kernel Parameters	10,180	4848	24,080	144	108
Largest Kernel Size	1 × 5	1 × 15	2 × 2	3 × 3	3 × 3
Method	CNN	ANN + CNN	CNN	bCNN	bDSCNN
Dataset	MIT-BIH	MIT-BIH	MIT-BIH	MIT-BIH	MIT-BIH
AAMI Standard	No	Yes	No	Yes	Yes
No. of MACs	${470,820}^{\;2}$	749,620	${12,823,040}^{\;2}$	129,969	${137,547 (122,697)}^{\;4}$
Multiplication Precision	float-32	float-32	float-32	1-bit	1-bit
Activation	ReLU	N/A	ReLU	bTanH	Binarized
${\mathrm{Acc}}_{N}$ (%)	$99.31^{\;3}$	98.59	$99.58^{\;3}$	N/A	96.96
${\mathrm{Acc}}_{S}$ (%)	N/A	99.10	$99.51^{\;3}$	N/A	98.50
${\mathrm{Acc}}_{V}$ (%)	$97.66^{\;3}$	99.40	$99.81^{\;3}$	97.30	98.73
${\mathrm{Acc}}_{F}$ (%)	N/A	99.70	N/A	N/A	99.60
${\mathrm{Acc}}_{Q}$ (%)	N/A	99.85	N/A	N/A	99.43
Output Classes	5	5	5	2	5

^1^ BP = better performance. ^2^ Estimated based on model parameters. ^3^ Classification accuracy in AAMI criteria. ^4^ Adopting the MCP method.

**Table 5:** FPGA performance evaluation of binarized-DSCNN-based heartbeat multi-classifier.

Type	TBioCAS 2020 [36]	NCA 2020 [70]	TBioCAS-BP 2021 [46]	TBioCAS-BP 2022 [47]	This Work
FPGA	Zynq XC7Z020	Artix7	iCE40UP5k	iCE40UP5k	DE1-SoC
Multiplication Precision	24-bit Fixed Point	24-bit Fixed Point	1-bit	1-bit	1-bit
Dataset	MIT-BIH	MIT-BIH	MIT-BIH	MIT-BIH	MIT-BIH
Network Type	MLP	MLP	bCNN	MLP + bCNN	bDSCNN
Additional Extractor Needed	Yes	Yes	No	No	No
No. of Input Samples	96	N/A	16 × 20	55	32 × 32
DSP Blocks	N/A	214	0	8	0
Hardware Resource	6600	9772	4977	6620	3799
Operating Clock (Hz)	2.5 M	98.2 M	100 K	100 K	100 K
Clock Cycles Per Classification	6298 *	N/A	1141	4794	3087
Dynamic Power (μW)	N/A	N/A	26	55	20
Energy Per Classification (nJ)	N/A	N/A	320.6	2839.1	617.4
Output Classes	5	2	2	2	5
${\mathrm{Acc}}_{V}$ (%)	99.6 **	95.0	97.3	98.5	98.7
${\mathrm{F1-score}}_{V}$ (%)	N/A	N/A	88.9	89.2	92.9
Acc (%)	99.7 **	N/A	N/A	N/A	96.6

* Calculated from the given data. ** The training data & the testing data are overlapped.

**Table 6:** Performance comparison between patient-specific and patient-wise dataset splitting methods.

Dataset Splitting Method	Acc (%)	No. of Kernels	No. of Kernel Parameters	No. of MACs	Hardware Resources	Clock Cycles per Classification	Energy per Classification (nJ)
Patient-specific	96.6	24	108	122,697	3799	3087	617
Patient-wise	92.1	28	120	146,357	3815	3785	757

## Data Availability

The data presented in this study are available on request from the corresponding author. The data are not publicly available due to other research works in progress using the same data.

## References

[1] WHO (2023). Noncommunicable Diseases. https://www.who.int/news-room/fact-sheets/detail/noncommunicable-diseases.

[2] WHO (2021). Cardiovascular Diseases (cvds). https://www.who.int/news-room/fact-sheets/detail/cardiovascular-diseases-(cvds).

[3] Meng L., Ge K., Song Y., Yang D., Lin Z. (2021). Long-term wearable electrocardiogram signal monitoring and analysis based on convolutional neural network. IEEE Trans. Instrum. Meas..

[4] Saadatnejad S., Oveisi M., Hashemi M. (2019). LSTM-based ECG classification for continuous monitoring on personal wearable devices. IEEE J. Biomed. Health Inform..

[5] Amirshahi A., Hashemi M. (2019). ECG classification algorithm based on STDP and R-STDP neural networks for real-time monitoring on ultra low-power personal wearable devices. IEEE Trans. Biomed. Circuits Syst..

[6] Luepker R.V., Raczynski J.M., Osganian S., Goldberg R.J., Finnegan J.R., Hedges J.R., Goff D.C., Eisenberg M.S., Zapka J.G., Feldman H.A. (2000). Effect of a community intervention on patient delay and emergency medical service use in acute coronary heart disease: The Rapid Early Action for Coronary Treatment (REACT) Trial. JAMA.

[7] Sadasivuni S., Damodaran V., Banerjee I., Sanyal A. Real-time prediction of cardiovascular diseases using reservoir-computing and fusion with electronic medical record. Proceedings of the 2022 IEEE 4th International Conference on Artificial Intelligence Circuits and Systems (AICAS).

[8] Djelouat H., Al Disi M., Boukhenoufa I., Amira A., Bensaali F., Kotronis C., Politi E., Nikolaidou M., Dimitrakopoulos G. (2020). Real-time ECG monitoring using compressive sensing on a heterogeneous multicore edge-device. Microprocess. Microsyst..

[9] John A., Panicker R.C., Cardiff B., Lian Y., John D. (2020). Binary classifiers for data integrity detection in wearable IoT edge devices. IEEE Open J. Circuits Syst..

[10] Martin T., Jovanov E., Raskovic D. Issues in wearable computing for medical monitoring applications: A case study of a wearable ECG monitoring device. Proceedings of the Digest of Papers. Fourth International Symposium on Wearable Computers.

[11] Zhang X., Lian Y. (2014). A 300-mV 220-nW event-driven ADC with real-time QRS detection for wearable ECG sensors. IEEE Trans. Biomed. Circuits Syst..

[12] Cheng Y., Lin M., Wu J., Zhu H., Shao X. (2021). Intelligent fault diagnosis of rotating machinery based on continuous wavelet transform-local binary convolutional neural network. Knowl.-Based Syst..

[13] Xian Z., Li H., Li Y. Weight Isolation-Based Binarized Neural Networks Accelerator. Proceedings of the 2020 IEEE International Symposium on Circuits and Systems (ISCAS).

[14] Yang Z., Zhou Q., Lei L., Zheng K., Xiang W. (2016). An IoT-cloud based wearable ECG monitoring system for smart healthcare. J. Med Syst..

[15] Wong D.L.T., Yu J., Li Y., Deepu C.J., Ngo D.H., Zhou C., Singh S.R., Koh A., Hong R., Veeravalli B. (2019). An integrated wearable wireless vital signs biosensor for continuous inpatient monitoring. IEEE Sens. J..

[16] Deepu C.J., Zhang X., Liew W.S., Wong D.L.T., Lian Y. (2014). An ECG-on-chip with 535 nW channel integrated lossless data compressor for wireless sensors. IEEE J. Solid-State Circuits.

[17] Deepu C.J., Xu X., Wong D., Heng C.H., Lian Y. (2018). A 2.3 *μ*W ECG-On-Chip for Wireless Wearable Sensors. IEEE Trans. Circuits Syst. II Express Briefs.

[18] Deepu C.J., Zhang X., Heng C.H., Lian Y. (2016). A 3-lead ECG-on-chip with QRS detection and lossless compression for wireless sensors. IEEE Trans. Circuits Syst. II Express Briefs.

[19] Diware S., Dash S., Gebregiorgis A., Joshi R.V., Strydis C., Hamdioui S., Bishnoi R. (2023). Severity-based hierarchical ECG classification using neural networks. IEEE Trans. Biomed. Circuits Syst..

[20] Pandey S., Voorsluys W., Niu S., Khandoker A., Buyya R. (2012). An autonomic cloud environment for hosting ECG data analysis services. Future Gener. Comput. Syst..

[21] El bouny L., Khalil M., Adib A. (2020). An end-to-end multi-level wavelet convolutional neural networks for heart diseases diagnosis. Neurocomputing.

[22] Fuster-Barceló C., Peris-Lopez P., Camara C. (2022). ELEKTRA: ELEKTRokardiomatrix application to biometric identification with convolutional neural networks. Neurocomputing.

[23] Kiranyaz S., Ince T., Gabbouj M. (2015). Real-time patient-specific ECG classification by 1-D convolutional neural networks. IEEE Trans. Biomed. Eng..

[24] Shyu L.Y., Wu Y.H., Hu W. (2004). Using wavelet transform and fuzzy neural network for VPC detection from the Holter ECG. IEEE Trans. Biomed. Eng..

[25] Wang N., Zhou J., Dai G., Huang J., Xie Y. (2019). Energy-efficient intelligent ECG monitoring for wearable devices. IEEE Trans. Biomed. Circuits Syst..

[26] Besler E., Mathur P.K., Gay H.C., Passman R.S., Sahakian A.V. (2021). Inter-patient atrial flutter classification using FFT-based features and a low-variance stacking classifier. IEEE Trans. Biomed. Eng..

[27] Salem M., Taheri S., Yuan J.S. ECG arrhythmia classification using transfer learning from 2-dimensional deep CNN features. Proceedings of the 2018 IEEE Biomedical Circuits and Systems Conference (BioCAS).

[28] Mao R., Li S., Zhang Z., Xia Z., Xiao J., Zhu Z., Liu J., Shan W., Chang L., Zhou J. (2022). An ultra-energy-efficient and high accuracy ECG classification processor with SNN inference assisted by on-chip ANN learning. IEEE Trans. Biomed. Circuits Syst..

[29] Li Y., Pang Y., Wang J., Li X. (2018). Patient-specific ECG classification by deeper CNN from generic to dedicated. Neurocomputing.

[30] Wang G., Chen M., Ding Z., Li J., Yang H., Zhang P. (2021). Inter-patient ECG arrhythmia heartbeat classification based on unsupervised domain adaptation. Neurocomputing.

[31] Sivapalan G., Nundy K.K., Dev S., Cardiff B., John D. (2022). ANNet: A lightweight neural network for ECG anomaly detection in IoT edge sensors. IEEE Trans. Biomed. Circuits Syst..

[32] Ullah A., Rehman S.U., Tu S., Mehmood R.M., Fawad, Ehatisham-ul Haq M. (2021). A hybrid deep CNN model for abnormal arrhythmia detection based on cardiac ECG signal. Sensors.

[33] Pandey S.K., Janghel R.R. (2019). Automatic detection of arrhythmia from imbalanced ECG database using CNN model with SMOTE. Australas. Phys. Eng. Sci. Med..

[34] Zhu F., Ye F., Fu Y., Liu Q., Shen B. (2019). Electrocardiogram generation with a bidirectional LSTM-CNN generative adversarial network. Sci. Rep..

[35] Chu H., Yan Y., Gan L., Jia H., Qian L., Huan Y., Zheng L., Zou Z. (2022). A neuromorphic processing system with spike-driven SNN processor for wearable ECG classification. IEEE Trans. Biomed. Circuits Syst..

[36] Zhao Y., Shang Z., Lian Y. (2019). A 13.34 μW event-driven patient-specific ANN cardiac arrhythmia classifier for wearable ECG sensors. IEEE Trans. Biomed. Circuits Syst..

[37] Abubakar S.M., Yin Y., Tan S., Jiang H., Wang Z. (2022). A 746 nW ECG processor ASIC based on ternary neural network. IEEE Trans. Biomed. Circuits Syst..

[38] Liang S., Yin S., Liu L., Luk W., Wei S. (2018). FP-BNN: Binarized neural network on FPGA. Neurocomputing.

[39] Saito T., Nonaka H., Okano T. (2024). Theoretical analysis of co-existing periodic orbits in sparse binary neural networks. Neurocomputing.

[40] Kim T.H., Shin J. (2020). A resource-efficient inference accelerator for binary convolutional neural networks. IEEE Trans. Circuits Syst. II Express Briefs.

[41] Liu Q., Lai J., Gao J. (2021). An efficient channel-aware sparse binarized neural networks inference accelerator. IEEE Trans. Circuits Syst. II Express Briefs.

[42] Kim H., Oh H., Kim J.J. Energy-efficient XNOR-free in-memory BNN accelerator with input distribution regularization. Proceedings of the 39th International Conference on Computer-Aided Design.

[43] Shreya S., Verma G., Piramanayagam S., Kaushik B.K. (2020). Energy-efficient all-spin BNN using voltage-controlled spin-orbit torque device for digit recognition. IEEE Trans. Electron Devices.

[44] Wang P., Song J., Peng Y., Liu G. Binarized neural network based on fpga to realize handwritten digit recognition. Proceedings of the 2020 IEEE International Conference on Information Technology, Big Data and Artificial Intelligence (ICIBA).

[45] Simons T., Lee D.J. (2019). A review of binarized neural networks. Electronics.

[46] Wong D.L.T., Li Y., John D., Ho W.K., Heng C.H. (2022). An energy efficient ECG ventricular ectopic beat classifier using binarized CNN for edge AI devices. IEEE Trans. Biomed. Circuits Syst..

[47] Wong D.L.T., Li Y., John D., Ho W.K., Heng C.H. (2022). Low complexity binarized 2D-CNN classifier for wearable edge ai devices. IEEE Trans. Biomed. Circuits Syst..

[48] Wong D.L.T., Li Y., John D., Ho W.K., Heng C.H. Resource and energy efficient implementation of ECG classifier using binarized CNN for edge AI devices. Proceedings of the 2021 IEEE International Symposium on Circuits and Systems (ISCAS).

[49] Sandler M., Howard A., Zhu M., Zhmoginov A., Chen L.C. Mobilenetv2: Inverted residuals and linear bottlenecks. Proceedings of the IEEE Conference on Computer Vision and Pattern Recognition.

[50] Bai L., Zhao Y., Huang X. (2018). A CNN accelerator on FPGA using depthwise separable convolution. IEEE Trans. Circuits Syst. II Express Briefs.

[51] Xuan L., Un K.F., Lam C.S., Martins R.P. (2022). An FPGA-based energy-efficient reconfigurable depthwise separable convolution accelerator for image recognition. IEEE Trans. Circuits Syst. II Express Briefs.

[52] Lu Y., Jiang M., Wei L., Zhang J., Wang Z., Wei B., Xia L. (2021). Automated arrhythmia classification using depthwise separable convolutional neural network with focal loss. Biomed. Signal Process. Control.

[53] Cai J., Sun W., Guan J., You I. (2020). Multi-ECGNet for ECG arrythmia multi-label classification. IEEE Access.

[54] Shan W., Yang M., Wang T., Lu Y., Cai H., Zhu L., Xu J., Wu C., Shi L., Yang J. (2020). A 510-nW wake-up keyword-spotting chip using serial-FFT-based MFCC and binarized depthwise separable CNN in 28-nm CMOS. IEEE J. Solid-State Circuits.

[55] Shan W., Yang M., Xu J., Lu Y., Zhang S., Wang T., Yang J., Shi L., Seok M. 14.1 A 510 nW 0.41 V low-memory low-computation keyword-spotting chip using serial FFT-based MFCC and binarized depthwise separable convolutional neural network in 28 nm CMOS. In Proceedings of the 2020 IEEE International Solid-State Circuits Conference-(ISSCC).

[56] Luo Y., Teng K.H., Li Y., Mao W., Lian Y., Heng C.H. (2019). A 74-*μ*W 11-Mb/s wireless vital signs monitoring SoC for three-lead ECG, respiration rate, and body temperature. IEEE Trans. Biomed. Circuits Syst..

[57] Zhang X., Zhang Z., Li Y., Liu C., Guo Y.X., Lian Y. (2016). A 2.89 *μ*W Dry-Electrode Enabled Clockless Wireless ECG SoC for Wearable Applications. IEEE J. Solid-State Circuits.

[58] Hsu S.Y., Ho Y., Chang P.Y., Su C., Lee C.Y. (2014). A 48.6-to-105.2 *μ*W machine learning assisted cardiac sensor SoC for mobile healthcare applications. IEEE J. Solid-State Circuits.

[59] Santurkar S., Tsipras D., Ilyas A., Madry A. (2018). How does batch normalization help optimization?. Adv. Neural Inf. Process. Syst..

[60] Chen T., Zhang Z., Ouyang X., Liu Z., Shen Z., Wang Z. “BNN-BN=?”: Training Binary Neural Networks Without Batch Normalization. Proceedings of the IEEE/CVF Conference on Computer Vision and Pattern Recognition.

[61] Ioffe S., Szegedy C. Batch normalization: Accelerating deep network training by reducing internal covariate shift. Proceedings of the International Conference on Machine Learning.

[62] Moody G.B., Mark R.G. (2001). The impact of the MIT-BIH arrhythmia database. IEEE Eng. Med. Biol. Mag..

[63] (1998). Testing and Reporting Performance Results of Cardiac Rhythm and ST Segment Measurement Algorithms.

[64] Acharya U.R., Oh S.L., Hagiwara Y., Tan J.H., Adam M., Gertych A., San Tan R. (2017). A deep convolutional neural network model to classify heartbeats. Comput. Biol. Med..

[65] Lu J., Liu D., Cheng X., Wei L., Hu A., Zou X. (2022). An efficient unstructured sparse convolutional neural network accelerator for wearable ECG classification device. IEEE Trans. Circuits Syst. I Regul. Pap..

[66] Lu J., Liu D., Liu Z., Cheng X., Wei L., Zhang C., Zou X., Liu B. (2021). Efficient hardware architecture of convolutional neural network for ECG classification in wearable healthcare device. IEEE Trans. Circuits Syst. I Regul. Pap..

[67] Jimenez-Perez G., Alcaine A., Camara O. (2020). ECG-DelNet: Delineation of ambulatory electrocardiograms with mixed quality labeling using neural networks. arXiv.

[68] Jimenez-Perez G., Acosta J., Alcaine A., Camara O. (2024). Generalising electrocardiogram detection and delineation: Training convolutional neural networks with synthetic data augmentation. Front. Cardiovasc. Med..

[69] Degirmenci M., Ozdemir M.A., Izci E., Akan A. (2022). Arrhythmic heartbeat classification using 2D convolutional neural networks. IRBM.

[70] Zairi H., Kedir Talha M., Meddah K., Ould Slimane S. (2020). FPGA-based system for artificial neural network arrhythmia classification. Neural Comput. Appl..

[71] Liu J., Zhu Z., Zhou Y., Wang N., Dai G., Liu Q., Xiao J., Xie Y., Zhong Z., Liu H. 4.5 BioAIP: A reconfigurable biomedical AI processor with adaptive learning for versatile intelligent health monitoring. In Proceedings of the 2021 IEEE International Solid-State Circuits Conference (ISSCC).

